# The LIFT trial: study protocol for a double-blind, randomised, placebo-controlled trial of K^+^-binder Lokelma for maximisation of RAAS inhibition in CKD patients with heart failure

**DOI:** 10.1186/s12882-021-02439-2

**Published:** 2021-07-06

**Authors:** Daniel Murphy, Irina Chis Ster, Juan-Carlos Kaski, Lisa Anderson, Debasish Banerjee

**Affiliations:** 1 St George’s University Hospitals NHS Foundation Trust, London, UK; 2 St George’s, University of London, Cranmer Terrace, London, SW17 0RE UK

**Keywords:** Chronic kidney disease, Heart failure, RAAS, Potassium binder, Randomised

## Abstract

**Background:**

CKD is common in heart failure (HF) and associated with morbidity and mortality, yet life-prolonging medications such as renin-angiotensin-aldosterone inhibitors (RAASi) are underused due to risk of hyperkalaemia. Sodium zirconium cyclosilicate (SZC) is a potassium-binding medication that has been shown to reduce incidence of hyperkalaemia in CKD, non-CKD, and HF populations, which we propose will support maximisation of RAASi therapy.

**Methods:**

We propose a 1:1 randomised, double-blind, placebo-controlled trial in which participants will receive either SZC or placebo. We will up-titrate participants’ RAASi therapy while monitoring their serum potassium levels and adjusting their SZC dose if necessary. Participants with CKD and HF will be recruited from CKD and HF clinics at St George’s Hospital. The total study period will be 18 months; 130 participants will be enrolled for approximately two months each following screening. Our primary outcome will be the proportion of participants who achieve maximum RAASi dose while maintaining normokalaemia. Secondary outcomes include participants reaching maximum RAASi dose without severe hyperkalaemia; time from randomisation to hyperkalaemia; time from randomisation to severe hyperkalaemia; number of RAASi dose escalations per participant; final doses of RAASi therapy; changes in quality of life score, eGFR, ACR, serum sodium, troponin T; number and duration of hospital admissions; and within-participant change in serum potassium compared to baseline.

**Discussion:**

This trial will be the first to examine the use of SZC for the maximisation of RAASi dosing in patients with advanced CKD and HF. We will assess the impact of achieving target RAASi dosing on hospital admission rates and duration of stay, with the hope that optimum RAASi treatment will translate into reduced morbidity and improved QoL. If clinical benefit is demonstrated, we hope that the joint multidisciplinary CKD-HF approach will be expanded.

**Trial registration:**

EudraCT number 2020–002946-18. Registered on 08 June 2020. Online record pending.

## Background

Chronic kidney disease (CKD) is a common comorbidity in individuals with heart failure (HF), with a prevalence of 32% according to a 2013 meta-analysis of over one million individuals [1]. Patients with combined CKD-HF are at 2.3 times greater risk of all-cause mortality than those with HF alone [1]. Potentially life-saving medications for systolic HF are underused in combined CKD-HF due to risk of hyperkalaemia [2]. These medications include renin-angiotensin-aldosterone system inhibitors (RAASi): angiotensin converting enzyme inhibitors (ACEi), angiotensin receptor blockers (ARB), and mineralocorticoid receptor antagonists (MRA) [3, 4].

While numerous national and international guidelines recommend RAASi treatment in patients with combined CKF-HF [5–8], the risk of hyperkalaemia in CKD stage 4/5 patients is high and potentially fatal [9]. There is therefore reluctance amongst general practitioners, general physicians, and cardiologists managing these patients to prescribe medications that may increase serum potassium levels [10].

In a study of 1056 acute heart failure admissions in 851 patients at St George’s Hospital, London, patients with CKD stage 4/5 and systolic HF were less likely to receive ACEi or ARB compared to non-CKD patients (36% vs 84%, *p* < 0.001) and less likely to receive MRA (17% vs 57%, *p* < 0.001) [11]. These findings have led to the establishment of a joint kidney failure-heart failure service at St George’s Hospital, funded by three local clinical commissioning groups, to improve care for these patients. Audit of all new referrals to the kidney failure-heart failure clinic between March and September 2017 showed that only 3% of all patients with left ventricular systolic dysfunction were on maximum-dose ACEi/ARB, and only 2% were on maximum-dose MRA [12].

Recent studies have shown that sodium zirconium cyclosilicate (SZC) can lower serum potassium concentrations in non-CKD, CKD, and HF populations [13–16]. SZC is a zirconium silicate compound that acts as a selective potassium ion trap and is not absorbed from the gastrointestinal tract [17]. In a phase three randomised controlled trial of 258 patients with hyperkalaemia, SZC lowered serum potassium from 5.6 to 4.5 mmol/L in 48 h and maintained serum potassium of < 5.1 mmol/L in 90% of patients (compared with 48% of patients on placebo, *p* < 0.001) [15].

Most studies of potassium binders have been short and none have investigated exclusively the high-risk group of patients with advanced CKD-HF, particularly with an aim to maximise RAASi therapy. Furthermore, this study will augment the currently-limited safety and efficacy data on RAASi maximisation in advanced CKD patients.

The St George’s experience suggests the need for a trial and subsequent clinical implementation of RAASi maximisation in patients with combined CKD-HF. There are significant numbers of patients in our own clinics who may benefit from recruitment into a trial of RAASi maximisation. We therefore propose a 1:1 randomised, double-blind, placebo-controlled trial to investigate the potential benefits of SZC in patients with CKD and HF on maximisation of RAASi therapy.

## Methods & design

### Study setting

Participants will be recruited from the joint kidney failure-heart failure clinic and from other heart failure clinics at St George’s Hospital. Patients discharged from the heart failure wards and inpatients admitted with acute heart failure and referred to the heart failure team will also be approached to take part. The study is being conducted in conjunction with St George’s, University of London and has been approved by the Cambridge NHS Research Ethics Committee [281626], NIHR portfolio [CPMS 47057] and the Medicines and Health Regulator Authority [CTA 12853/0009/001-0001].

### Eligibility criteria

#### Inclusion criteria

For inclusion in the study patients must fulfil all of the following criteria:

1. Age > 18 years
2. Heart failure, clinical or echo confirmed (HFrEF i.e. ejection fraction < 40%); patients with atrial fibrillation will be included provided the EF can be determined
3. New York Heart Association class II to IV
4. Most recent serum potassium 5.0–5.5 mmol/L
5. Adequate blood pressure (BP) (> 90 mmHg systolic and without postural hypotension; drop of systolic blood pressure (SBP) > 20 mmHg or feeling dizzy with change in posture; exclude patients with symptomatic hypotension due to high doses of ACEi/ARB or MRA unless the clinical condition has improved)
6. Formal diagnosis of CKD (i.e. two measurements of eGFR < 60 mL/min/1.73 m^2^ taken ≥3 months apart) with stable eGFR < 60 mL/min/1.73m^2^. eGFR to be calculated according to the CKD-Epi formula [18], measured at a single time-point.
7. None or submaximal dose of ACEi/ARB and/or MRA.

#### Exclusion criteria

Patients should not enter the study if any of the following exclusion criteria are fulfilled:

Pregnancy: individuals of childbearing potential must have a negative pregnancy test within 7 days prior to treatment initiation and agree to use highly-effective contraception for the trial period

1. Active malignancy or infection
2. BMI > 35 kg/m^2^
3. Poorly controlled blood sugar (HbA1c > 70 mmol/mmol)
4. Recent Acute Coronary Syndrome (ACS) i.e. within three months
5. Ongoing potassium therapy
6. Prolonged QT >  550 ms, congenital QT syndrome and history of prolonged QT requiring drug discontinuation
7. Currently breastfeeding
8. Allergies to excipients of investigational medicinal product (IMP) or placebo
9. Inability to consent

### Recruitment

Participants will be identified by the research team from the clinics mentioned above by consulting clinic lists and patients’ blood test results. Potential participants nearing discharged from hospital will be referred to the research team for participation, provided they are stable and would benefit from RAASi maximisation. Eligibility for the study in all cases will be confirmed by the Principal Investigator (PI) or another study doctor. All patients who fit the eligibility criteria for the study will be approached. At this initial contact, potential participants will be informed of the nature and objectives of the study and any potential risks of participation. If participants agree to take part, they will be invited for screening.

At screening, all participants will have their last three months’ blood tests results for potassium and creatinine reviewed. An electrocardiogram (ECG) will be performed to record participants’ QT interval and further blood samples will be taken for analysis (see *Study Timeline* below). These tests may be done as part of standard care if clinically necessary. Participants of childbearing potential will be required to take a urine pregnancy test. Eligible participants will be asked to complete an E5-QD quality-of-life (QoL) questionnaire and will receive educational material on low sodium / low potassium diets. Again, the PI or study doctor will confirm eligibility in all cases.

Full informed consent will be obtained prior to a participant undergoing any study activities. All potential participants will undergo assessment of capacity, with the opportunity for potential participants to ask questions and have a period of at least 24 h to consider if they would like to take part. The study team will assess if the participant is able to:

1. Understand the purpose and nature of the research;
2. Understand what the research involves, its benefits, risks and burdens;
3. Understand the alternatives to taking part;
4. Retain the information long enough to make an effective decision;
5. Be able to make a free choice;
6. Be capable of making this particular decision at the time it needs to be made (though their capacity may fluctuate, and they may be capable of making some decisions but not others depending on their complexity).

If the above criteria are met, written consent may be taken by the PI or study doctor. All participants may refuse or discontinue involvement at any time without giving a reason for doing so.

### Randomisation

Participants will be randomised 1:1 to either the placebo or active (SZC) arm of the trial. Randomisation is stratified by patients’ baseline RAASi dose at the start of the trial (individuals on < 50% standard dose vs those on ≥50% standard dose) to ensure equal proportions of RAASi therapies in each group.

We will use the REDCap Randomisation Module (https://wiki.uiowa.edu/display/REDCapDocs/REDCap+Randomization+Module). This module can be turned on by selecting the “Enable the randomization module” checkbox during project creation or after. This would preserve the randomisation procedure independent of the study statistician.

Randomisation will be implemented by the research pharmacy at St George’s. Randomisation codes will be generated by the study statistician on an external spreadsheet. The research pharmacy will be informed by the study team as soon the patient is consented, and will use the randomisation list to assign the participant to their allocated arm. In the case of an adverse event or patient safety issue, emergency unblinding will be performed by the on-call research pharmacist.

### Confidentiality

The study will be carried out in accordance with Good Clinical Practice guidance and UK data regulation legislation. All participants will be given an anonymous study ID, which will be used for all data and samples collected. Participants’ personal details will be stored in paper files in a locked office, access to which will be restricted to necessary personnel. Study team members looking at data collected or test results will not be able to see participants’ personal details. All personal information will be destroyed one year following the end of the study.

### Risk in the context of the COVID-19 pandemic

In the context of the COVID-19 pandemic, one potential safety concern is that required two-weekly study appointments may increase the risk of participants being exposed to SARS-CoV-2. A COVID-19 risk assessment has been performed, all study personnel will be instructed and trained in infection control measures and, where possible, social distancing practises will be observed.

### Study timeline

**Fig. 1:**
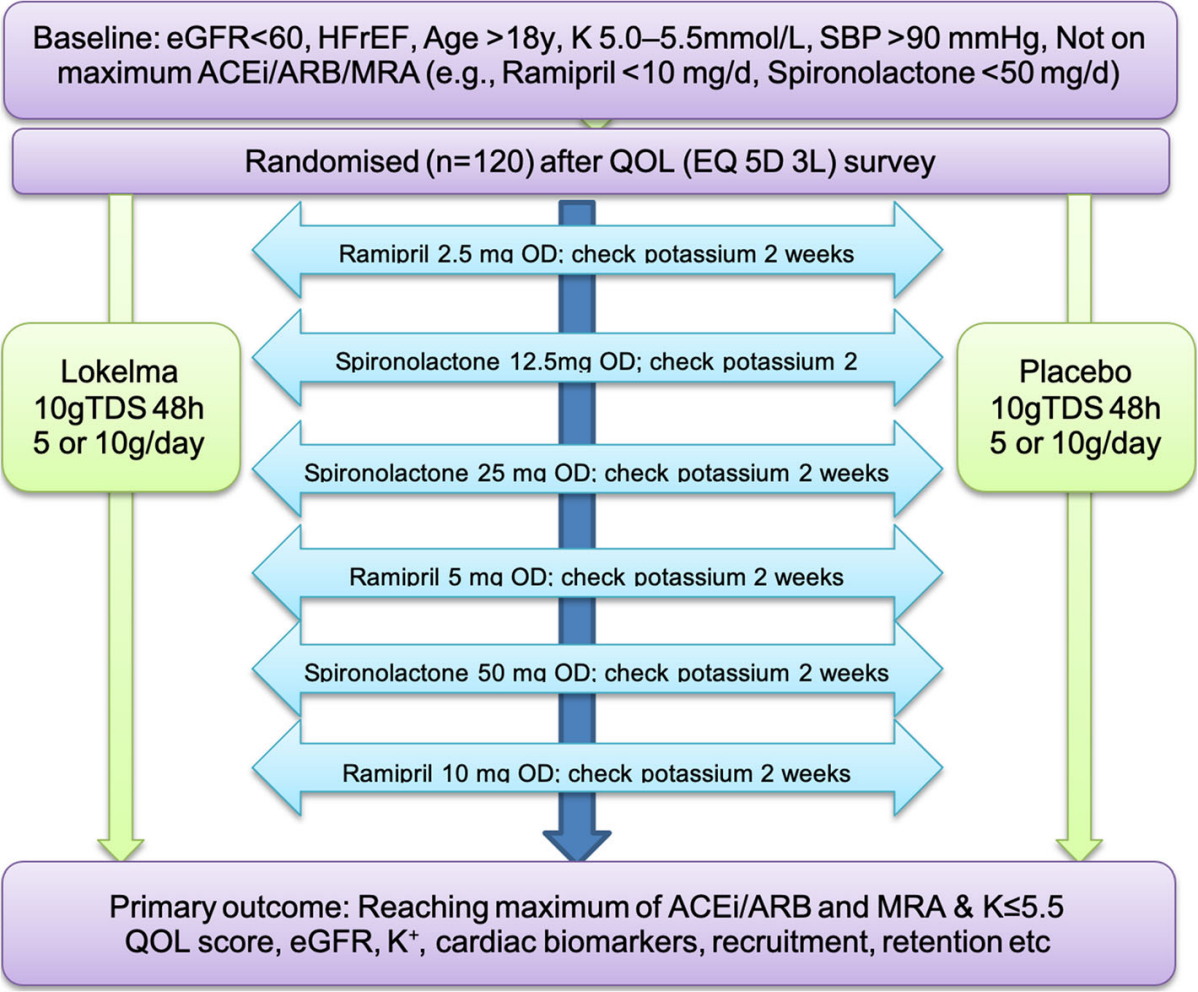
Study flow chart. Flow chart demonstrating design of the study. ACEi = ACE inhibitor, ARB = angiotensin receptor blocker, eGFR = estimated glomerular filtration rate, HFrEF = heart failure with reduced ejection fraction, MRA = mineralocorticoid receptor antagonist, OD = once daily, QOL = quality of life, SBP = systolic blood pressure, TDS = three times daily

The study activity schedule is outlined in Table 1 and represented schematically in Fig. 1. Baseline data will be recorded on electronic case report forms (eCRFs) and will include demographic information (age, gender, ethnicity), clinical co-morbidities (e.g. diabetes, hypertension, ischaemic heart disease), cardiovascular risk factors (e.g. hypercholesterolaemia, BMI, smoking, family history of cardiac disease), blood test results (e.g. serum haemoglobin, ferritin, urea, creatinine, sodium, potassium, calcium, phosphate, B-type natriuretic protein [BNP], troponin, parathyroid hormone [PTH]), ECG, and echocardiogram results. In addition, we will record pulse rate, blood pressure (average of three readings), weight, presence of oedema, presence of chest crepitations, signs of raised jugular venous pressure, Rockwood clinical frailty score, and E5-QD QoL questionnaire score. The end-of-trial visit will record the same data.

**Table 1:** Study activity schedule and procedures

	Screening	C1	C2	C3	C4	C5	C6	C7 or final visit
Week	0	0	Every 2 weeks ±2 days					
Day (approx.)	1	1	3	17	31	45	59	59 or 73
**Informed Consent**								
Informed consent	X							
**Study procedures**								
Targeted physical exam (based on symptoms)	X	X	X	X	X	X	X	X
Vital signs	X	X	X	X	X	X	X	X
Past medical history								
ECG	X		X					X
Concomitant medications	X							
Demographics	X							
Eligibility criteria	X							
A:CR	X							
**Laboratory assessments**								
Baseline blood tests	X							
iSTAT sK	X	X	X	X	X	X	X	X
Lab serum K	X	X	X	X	X	X	X	X
Laboratory safety assessment		X	X	X	X	X	X	X
**Monitoring**								
AE/SAE assessment	Ongoing							
**Drug dosing and monitoring**								
LOKELMA/Placebo		X	X	X	X	X	X	X
RAASi		X	X	X	X	X	X	X
Diuretics use	X	X	X	X	X	X	X	X
**Efficacy evaluations/Other assessments and assays**								
Quality of Life assessment	X							X
Hospitalization assessment		X	X	X	X	X	X	X
HF symptom assessment		X	X	X	X	X	X	X

Table 1—Study schedule of procedures. C6 (i.e. 7 visits total) will occur only in participants not prescribed RAASi at study entry. Baseline blood tests: serum haemoglobin, ferritin, urea, creatinine, sodium, potassium, BNP, troponin, calcium, phosphate, PTH. Laboratory safety assessment: serum sodium, potassium, creatinine

At initial visit, participants will be initiated on placebo or SZC at initiation dose (i.e. 10 g three times daily). If participants are already taking RAASi medication, they will continue their current medication. If not, the study doctor will start them on appropriate RAASi therapy per local guidance. Participants will be reviewed at 48 h where they will have serum potassium concentration measured and enter the maintenance phase. If serum potassium is 3.5–5.0 mmol/L, participants will begin standard maintenance-dose SZC (i.e. 5 g once daily). For other serum potassium concentrations, and for the remainder of the maintenance phase, dosing will be titrated as outlined in Table 2. If participants experience other adverse effects from RAASi therapy (e.g. symptomatic hypotension, < 90 mmHg recorded systolic blood pressure, postural hypotension), their RAASi dose will be down-titrated if clinically indicated.

**Table 2:** IMP dosage protocol

SZC dose at start of visit	Serum potassium at visit	Dose change at end of visit
On 10 g daily	5.0–6.0 mmol/L	Continue 10 g daily
	3.5–5.0 mmol/L	Change to 5 g daily
	< 3.5 mmol/L	Stop
	> 6.0 mmol/L	Stop and initiate rescue
On 5 g daily	> 5.0 mmol/L	Change to 10 g daily
	3.5–5.0 mmol/L	Continue 5 g daily
	< 3.5 mmol/L	Stop
On 0 g daily (i.e. temporary stop)	≤ 5.0 mmol/L	Continue with no IMP
	> 5.0 mmol/L	Initiate 5 g daily

Table 2—Dosage protocol for study visits based on serum potassium measurements. IMP may be restarted two weeks after discontinuation due to hypokalaemia depending on serum potassium result

After screening, recruitment, and randomisation, visits will take place every two weeks (± two days) until the end of the study. The number of visits will depend on participants’ baseline dose of ACEi / ARB / MRA. Patients with no prescribed RAASi at baseline will have seven visits, and others will have six visits.

Following the final study visit, there will be no further follow up visits related to the trial.

### Sample collection and analysis

Serum samples will be obtained by venepuncture by one of the research team. Samples for potassium, sodium, calcium, phosphate, urea, creatinine, ferritin, troponin T and BNP testing will be collected into 5 mL SST tubes. Samples for haemoglobin and PTH testing will be collected into 4 mL EDTA tubes. All analysis will be carried out at the NHS South West London Pathology laboratory at St George’s Hospital. With participant consent, serum samples will be saved and stored in a − 80 °C freezer in the Cardiovascular Research Institute at St George’s for the duration of the study.

### IMP and placebo information

The unlabelled study drug (SZC, brand name Lokelma®) or placebo will be provided by AstraZeneca PLC (Cambridge, UK) and delivered to St George’s research pharmacy in bulk. Lokelma® is licensed for use in the UK and European Union for the treatment of hyperkalaemia. The study drug will be labelled upon receipt by an independent company (RenaClinical, Horley, UK) and prepared in accordance with Good Manufacturing Practice and local regulatory guidance.

The study drug will be supplied as a powder for oral suspension in 5 g or 10 g sachets. The entire contents of the sachet should be emptied into a drinking glass containing approximately 45 mL of water and stirred well. The powder does not dissolve, and the tasteless liquid should be consumed while still cloudy. The study drug does not require any special storage conditions, can be taken with or without food, and can be taken with many other medications.

### Outcomes

The main clinical outcome measure will be serum potassium concentration, which will be measured every two weeks, and the dose of RAASi at the end of the study. The statistical outcome measure will be achieving target level of RAASi without hyperkalaemia (i.e. serum potassium ≥5.6 mmol/L). Target RAASi doses are defined as maximum dosing as in Table 3. Outcomes are also displayed in Table 4.

**Table 3:** Dose classification of RAASi therapy

	Minimum dose	Half maximum dose	Maximum dose
**ACEi**			
Ramipril	2.5 mg OD	5 mg OD	10 mg OD
Perindopril arginine	2.5 mg OD	5 mg OD	10 mg OD
Enalapril	2.5 mg OD	5 mg OD	10 / 20 / 40 mg OD
Lisinopril	2.5 mg OD	5 / 10 mg OD	20 / 40 mg OD
Perindopril erbumine	2 mg OD	4 mg OD	8 / 16 mg OD
**ARB**			
Losartan	25 mg OD	50 mg OD	100 mg OD
Candesartan	4 mg OD	8 / 16 mg OD	32 mg OD
Irbesartan	75 mg OD	150 mg OD	300 mg OD
Telmisartan	20 mg OD	40 mg OD	80 mg OD
Olmesartan	10 mg OD	20 mg OD	40 mg OD
Valsartan	40 mg BD	80 mg BD	160 mg BD
**MRA**			
Spironolactone	12.5 mg OD	25 mg OD	50 mg OD
Eplerenone	12.5 mg OD	25 mg OD	50 mg OD
**ARNI**			
Valsartan + sacubitril	24 / 26 mg BD	49 / 51 mg BD	97 / 103 mg BD

Table 3—ARNI = angiotensin receptor-neprilysin inhibitor, BD = twice daily, OD = once daily. For analysis and dose titration, participants’ RAASi doses will be classified as low / medium / maximum based on the values in this table. Note—this is not a dose conversion table

**Table 4:** Primary and secondary study outcomes

Objectives	Outcome measures	Timepoints of evaluation
**Primary Objective:** To compare SZC vs placebo with respect to:	Enabling participants to achieve target RAASi dose while keeping serum potassium < 5.6 mmol/L.	Blood test for potassium and RAASi dose
**Secondary Objectives:** To compare SZC vs placebo with respect to:	Achieving maximum dose of MRA and ACEi/ARB with serum potassium < 5.6 mmol/L Achieving maximum dose of MRA and ACEi/ARB with serum potasssium < 6.0 mmol/L Time since randomisation to first occurrence of hyperkalaemia (serum potassium ≥5.6 mmol/L) Time since randomisation to severe hyperkalaemia (serum potassium ≥6.0 mmol/L) Number of ACEi/ARB, MRA dose changes Number and durationof hospital admissions during the study Change in serum potassium at respective visits vs baseline	Blood test for potassium and RAASi dose ever two weeks. Other parameters checked at baseline and end of study.
**Tertiary Objectives:**	None	

Table 4—Primary and secondary study outcomes

#### Primary outcomes

Participants will be classified as either responders or non-responders, where a responder is a participant who achieves their target RAASi dose with no post-randomisation serum potassium measurements ≥5.6 mmol/L. Participants who discontinue the study before completion will be deemed as missing.

#### Secondary outcomes

1. Responder / non-responder classification, where a responder is defined as a patient who achieved maximum dose of MRA *and* ACEi/ARB with serum potassium ≤5.6 mmol/L;
2. Responder / non-responder classification, where a responder is defined as a patient who achieved maximum dose of MRA and or ACEi/ARB and serum potassium ≤6.0 mmol/L;
3. Time from randomisation to hyperkalaemia (serum potassium ≥5.6 mmol/L);
4. Time from randomisation to severe hyperkalaemia (serum potassium > 6.0 mmol/L);
5. Number of RAASi dose escalations per patient;
6. Final doses of ACEi/ARB, MRA;
7. Change in QoL score, eGFR, ACR, BNP, serum sodium, troponin T at event / end as compared to baseline;
8. Number of hospital admissions per patient, as well as duration of hospital admissions;
9. Within-patient change in serum potassium as compared to baseline;

### Statistical analysis and sample size

#### Summary of baseline data and flow of patients

We will perform an intention-to-treat analysis. The primary outcome (i.e. responder / non-responder status) will be analysed via χ^2^ test with α = 0.05. The proportion of responders in each arm, the difference thereof, and the 95% confidence interval for the difference in proportions will be reported.

Additional analyses may be performed to explore the consistency of the effect across subgroups, for instance defined by the degree of MRA and/or ACEi/ARB treatment at baseline. Provided the modelling assumptions are satisfied (e.g. sufficient numbers of responders / non-responders in each arm), these will be done by logistic regression.

If there is loss to follow-up, sensitivity analysis will be conducted. Given the binary nature of the primary outcome, extreme scenarios such as the worst case in the intervention group (all losses to follow-up classified as non-responders) and the best case in the control group (all losses to follow-up are responders) will be considered.

#### Secondary outcome analysis

Descriptive summaries of the distribution of the secondary endpoints will be presented. More formal statistical analysis and modelling may also be performed: for responder/non-responder outcome (e.g. secondary outcomes 1 and 2), analysis may be done in the same manner as for the primary outcome. For time-to-event endpoints (e.g. secondary outcomes 3 and 4), survival techniques such as Kaplan-Meier estimates may be employed. The exact choice of analyses will be data-driven, so these analyses are to be regarded as explanatory.

#### Sample size estimation

Clinical evidence suggests that the proportion of patients reaching the target dose of RAASi in the control arm would be around 50% as a conservative estimation based on our experience. The study is powered to detect an effect size of the intervention of around 30%.

Group sample sizes of 52 will achieve approximately 90% power to detect a difference between the group proportions of 0.3 using a two-sided Z-test with pooled variance and a two-sided χ^2^ test. The proportion of responders in the placebo arm is assumed to be 0.5 with the corresponding proportion in the active arm 0.8 under the alternative hypothesis. The significance level (α) of the test is 0.05. This results in a total of 104 patients required and, accounting for 20% loss to follow-up, we will recruit 130 patients to be randomised to each group.

#### Interim analysis and criteria for trial termination

An interim analysis reviewing feasibility and tolerability will be performed once 10% of patients have completed the study. We envisage a further two interim analyses, upon collection of 50 and 75% of events. No early conclusions will be drawn until data collection is completed and the data is locked.

#### Procedure(s) to account for missing or spurious data

If data exhibit missing or spurious values, the circumstances of their collection, recording, and analysis will be investigated. Appropriate statistical analysis would be carried out depending on the type of data missing (i.e. independent or dependent variables).

### Data management and security

Data will be collected into eCRFs and accessible to members of the study team by a unique username-password combination. Participants’ clinical data is stored securely by St George’s University Hospitals NHS Foundation Trust. Access will be granted to authorised representatives of the Sponsor, host institution, and regulatory authorities to permit trial-related monitoring, audit, and inspection in line with participant consent. Data management will comply with EU General Data Protection Regulation (2018) and the UK Data Protection Act (2018).

### Dissemination policy

The Chief Investigator will liaise with all investigators to compile data and submit a manuscript for peer review with a view to publication in a reputable scientific journal within 180 days of study completion (i.e. the “Main Publication”). Following the Main Publication, other investigators may prepare further publications. Results from the study will also be communicated to trial participants and local and national patients’ associations.

## Discussion

This trial will be the first to look at the use of SZC for the maximisation of RAASi dosing in CKD patients. Our hypothesis is that SZC therapy will reduce the incidence of treatment-limiting hyperkalaemia in CKD-HF patients, allowing higher doses of RAASi therapy and therefore better clinical outcomes. Achievement of the participant’s target RAASi dose is the primary endpoint, and secondary endpoints include measures to assess safety in terms of hyperkalaemia, cardiovascular risk in terms of troponin T levels [19, 20], and efficacy in terms of time-to-target dose. We will also assess the impact of achieving target RAASi dosing on hospital admission rates and duration of stay, with the hope that optimum RAASi treatment will translate into reduced morbidity and improved QoL.

The strengths of the study include its randomised, double-blind design, and the favourable safety profile of the trial drug. We intend that the design will provide high-quality evidence that, if our hypothesis is supported, will help guide changes in clinical practise. As SZC is approved for the treatment of hyperkalaemia in the UK and has a favourable safety profile, our study has the benefit of being low-risk to participants.

The side-effect profile of SZC is minimal and hence we hope that adherence to the study protocol will be satisfactory. Our statistical predictions have nonetheless accounted for a 20% dropout rate owing to participants’ decisions, movements out of the area, death, progression to renal transplant, or other causes. Our study design focusses on a single recruitment centre.

The novel value of this study centres around (I) its examinations of SZC to limit hyperkalaemia and allow RAASi maximisation in the under-studied combined CKD-HF patient group, and (ii) its expansion of the currently limited safety data on the use of RAASi therapy in advanced CKD patients. The joint CKD-HF clinic approach is an emerging practise, and, if clinical benefit is proven, we would hope to investigate future studies with wider scope for more extensive population validity.

## Data Availability

There are currently no plans to release raw data generated by the study..

## Abbreviations

ACEi: angiotensin converting enzyme inhibitors
ACR: albumin-creatinine ratio
AE: adverse event
ARB: angiotensin receptor blockers
ARNI: angiotensin receptor-neprilysin inhibitors
BNP: B-type natriuretic peptide
CKD: chronic kidney disease
ECG: electrocardiogram
eCRF: electronic case report form
eGFR: estimated glomerular filtration rate
HF: heart failure
IMP: investigational medicinal product
MRA: mineralocorticoid receptor antagonists
PTH: parathyroid hormone
QoL: quality of life
RAASi: renin-angiotensin-aldosterone system inhibitors
SAE: suspected adverse event
SZC: sodium zirconium cyclosilitate
